# Molecular Genetics of 1α-Hydroxylase Deficiency in the Saudi Population

**DOI:** 10.1210/jendso/bvaf183

**Published:** 2025-11-17

**Authors:** Bassam Bin-Abbas, Afaf Alsagheir, Balgees Alghamdi, Allianah Benito, Yufei Shi, Somaya Khader Alzelaye, Noman Ahmad, Fahad Al Juraibah, Amer Omar Alali, Adnan Al Shaikh, Najya Attia, Abdulhameed Abdulmohsen Albunyan, Abdullah Saad Alshahrany, Ahmed Ali Nahari, Nabilah Sulaimani, Khloud M Alrubaya, Ali S Alzahrani

**Affiliations:** Section of Pediatric Endocrinology, Department of Pediatrics, King Faisal Specialist Hospital & Research Centre, Riyadh 11211, Saudi Arabia; Section of Pediatric Endocrinology, Department of Pediatrics, King Faisal Specialist Hospital & Research Centre, Riyadh 11211, Saudi Arabia; Department of Molecular Oncology, Research and Innovation, King Faisal Specialist Hospital and Research Centre, Riyadh 11211, Saudi Arabia; Department of Molecular Oncology, Research and Innovation, King Faisal Specialist Hospital and Research Centre, Riyadh 11211, Saudi Arabia; Department of Molecular Oncology, Research and Innovation, King Faisal Specialist Hospital and Research Centre, Riyadh 11211, Saudi Arabia; Centre of Endocrinology and Diabetes Mellitus, Al-Qunfudah General Hospital, Al-Qunfudah 28814, Saudi Arabia; Department of Pediatrics, King Faisal Specialist Hospital and Research Centre, Jeddah 23433, Saudi Arabia; College of Medicine, King Saud bin Abdul-Aziz University for Health Sciences, Ministry of National Guard Health Affairs, Riyadh 11481, Saudi Arabia; Department of Pediatrics, Jazan Hospital, Jazan 82874, Saudi Arabia; Department of Pediatrics, King Abdulaziz Medical City (KAMC), King Saud Bin Abdulaziz University for Health Sciences (KSAU-HS), King Abdullah International Medical Research Centre (KAIMRC), Ministry of National Guard Health Affairs (MNGHA), Jeddah 21423, Saudi Arabia; Department of Pediatrics, King Abdulaziz Medical City (KAMC), King Saud Bin Abdulaziz University for Health Sciences (KSAU-HS), King Abdullah International Medical Research Centre (KAIMRC), Ministry of National Guard Health Affairs (MNGHA), Jeddah 21423, Saudi Arabia; Department of Paediatrics, MOH-Saudi Arabia, Maternity and Children Hospital Alhasa-Hofuf 36422, Saudi Arabia; Department of Pediatrics, Armed Forces Hospital Southern Region, Khamyes Mushait 62413, Saudi Arabia; Department of Pediatrics, Jazan Specialist Hospital, Jazan 45142, Saudi Arabia; Department of Pediatrics,Maternity and Children Hospital–Makkah, Makkah 24246, Saudi Arabia; Department of Pediatrics, Maternity and Children Hospital–Dammam, Dammam 32253, Saudi Arabia; Department of Molecular Oncology, Research and Innovation, King Faisal Specialist Hospital and Research Centre, Riyadh 11211, Saudi Arabia; Department of Medicine, King Faisal Specialist Hospital & Research Centre, Riyadh 11211, Saudi Arabia

**Keywords:** 1α-hydroxylase, *CYP27B1*, vitamin D–resistant rickets type 1A, vitamin D–dependent rickets

## Abstract

**Context:**

The aim of this study was to characterize the molecular genetics of 1α-hydroxylase deficiency in the highly consanguineous population of Saudi Arabia, hypothesizing that the results will show a unique *CYP27B1* genotype.

**Methods:**

We collected data on a large cohort of patients diagnosed with 1α-hydroxylase deficiency from different parts of the country. These patients underwent molecular testing for *CYP27B1* mutations.

**Results:**

A cohort of 45 patients from 29 unrelated families was studied. In 13 families (29 patients), more than one affected sibling was included (2-4 siblings) while the other 16 families had only a single patient per family. The patients included 24 females and 21 males with median age at time of presentation of 1 year and a current median age of 10 years. The clinical, biochemical and radiological profile was typical of 1α-hydroxylase deficiency. Molecular testing showed 10 mutations of different types in the 29 families. Four mutations were novel (p.(Trp257LeufsTer76), p.(Glu101Gln), p.(Gly398Ser), and p.(Arg206Cys)) while the other 6 mutations were previously described (p.(arg429Pro), p.(Phe443Profs*24), p.(Gln135Ter), p. (Gly102Glu), p.(Gln504Ter), and c.589 + 1G > A). Two of the previously reported mutations were from Saudi patients and have never been reported from other populations, increasing the number of novel/previously novel mutations to 6 of 10 mutations (60%). The most common mutation was c.1286G > C, p.(Arg429Pro) occurring in 22 patients from 15 unrelated families (51.7%).

**Conclusion:**

The molecular genetics of 1α-hydroxylase deficiency in Saudi Arabia is unique with several novel mutations of different types and a possible founder mutation.

Vitamin D is one of the most important vitamins and is essential for skeletal development and health. It also has important functions in the immune system, muscle activity, and many other cellular processes [1]. In humans, vitamin D exists in 2 forms, vitamin D2 (ergocalciferol) and D3 (cholecalciferol) [1]. The body normally obtains vitamin D2 from plant sources such as mushrooms and fortified foods through the gastrointestinal tract [1, 2]. Vitamin D3 is obtained from animal sources such as fish, egg yolks, liver, and fortified dairy products but is mainly synthesized in the skin from conversion of 7-dehydrocholesterol under the activation by solar ultraviolet light [1, 2]. Vitamin D2 and D3 are both inactive prohormones that need a 2-step hydroxylation for their activation. The first step is the hepatic 25-hydroxylation where cholecalciferol is converted to 25-hydroxycholecalciferol by several enzymes that have 25-hydroxylase activity such as the 2 microsomal enzymes, CYP2R1 and CYP3A4 and the mitochondrial enzyme CYP27A1 [3-5]. 25-OH Vitamin D needs a second hydroxylation step for full activation. That occurs mainly in the kidney under the influence of the mitochondrial 1α-hydroxylase (CYP27B1) [1, 6]. This rate-limiting step results in the conversion of 25-OH vitamin D to the active 1, 25 dihydroxycholecalciferol (1, 25-(OH)_2_D). (1, 25-(OH)_2_D) exerts its actions mainly on the gastrointestinal tract through its nuclear receptor (VDR) [1, 5, 6]. Activation of VDR results in increased absorption of calcium and phosphorous [1, 5, 6]. In addition to its central role in calcium and phosphorus homeostasis, (1, 25-(OH)_2_D) has other roles in cell proliferation and differentiation of a variety of tissues [1, 5, 6]. CYP27B1 activity is regulated by the parathyroid hormone, serum calcium, and phosphorous level and the fibroblast growth factor-23 [1, 6, 7].

1α-Hydroxylase deficiency results in vitamin D–dependent rickets type 1A (VDDR-IA), a rare autosomal recessive disorder caused by *CYP27B1* mutations [5, 6, 8]. It usually presents in infancy and early childhood with hypotonia, growth retardation, and muscle weakness, and radiological changes of rickets that, if untreated, progress to bone deformities [6-8]. Biochemically, patients usually have hypocalcemia, hypophosphatemia, high alkaline phosphatase, and secondary hyperparathyroidism [5, 7]. More than 100 *CYP27B1* mutations have been reported in the Human Gene Mutation Database from different ethnic groups (accessed June 25, 2025) [9]. There are some ethnic differences in the type and distribution of these mutations [10-12].

Saudi Arabia has a high rate of consanguinity [13, 14]. Studies have reported consanguinity rates of 50% to 67% [13, 15], with regional variations reaching in some areas to 80% [13, 15, 16]. First-cousin marriages are the most common, accounting for 25% to 35% [13, 15, 17]. This increases the risk of autosomal recessive disorders such as 1α-hydroxylase deficiency. A few previous studies suggested that the molecular profile of 1α-hydroxylase (*CYP27B1*) deficiency is unique in Saudi Arabia [18-22]. However, all previous reports are in the form of case reports or single families. In this study, our objective was to study the molecular genetics of this hereditary condition in the highly consanguineous population of Saudi Arabia, hypothesizing that the results will show a unique molecular profile. Therefore, we studied a large cohort of patients consisting of 45 patients from 29 families collected from different parts of the country.

## Materials and Methods

After obtaining an institutional review board approval from our institutional review board (RAC No. 2130-012, King Faisal Specialist Hospital and Research Centre, Riyadh, Saudi Arabia), we retrospectively collected clinical and biochemical data and genetic testing data on patients with an established diagnosis of 1α-hydroxylase deficiency (*CYP27B1* mutations). All genetic tests were performed by accredited molecular testing laboratories (Centogene). We verified the reported mutations by Sanger sequencing in the majority of cases. The DNA isolation from peripheral leucocytes, primers, polymerase chain reaction, and sequencing conditions are as previously reported [19].

## Results

We studied 45 patients from 29 families; 29 of them came from 13 unrelated families with 2 to 4 members affected, and 16 patients were single patients from the remaining 16 unrelated families. All patients and their families are from the endogenous native population of Saudi Arabia and none was an immigrant.

### Clinical Data

The clinical, biochemical, and radiological profiles of each patient are summarized in Supplementary Table S1 [23]. The cohort includes 24 females (53.3%) and 21 males (46.7%). Patients presented generally between ages 2 months and 3 years (median age at presentation was 1 year). Current age ranges between 1 and 32 years (median 10 years). Common symptoms included delayed walking, hypotonia, bowing of legs, wrist widening, and short stature. The hallmarks of the biochemical profile were low to low-normal serum calcium level (some patients were already on treatment at the time of evaluation), low normal to low phosphorous, very high alkaline phosphatase, and secondary hyperparathyroidism. Radiologically, most patients had the typical signs of rickets with cupping and fraying of metaphysis and some had bowing of the legs. Patients were treated with alfacalcidol or calcitriol and calcium supplements.

### Molecular Data

**Figure 1.**
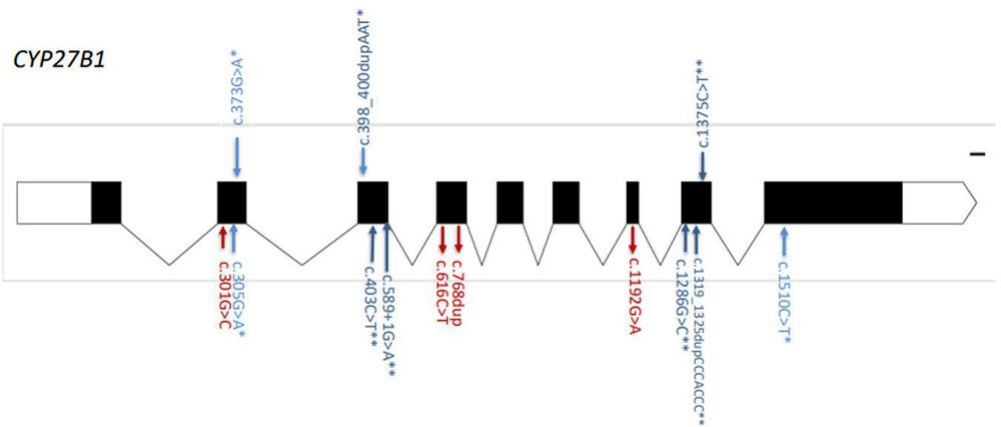
*CYP27B1* gene sketch showing the 9 exons (solid rectangles) and 8 introns (connecting lines) with mutations found in this study plotted on the bottom and those previously reported from Saudi Arabia plotted above. The red color mutations are novel, the blue ones reported before, and the ones with an asterisk (*) were reported only once from Saudi Arabia and (**) reported from Saudi and/or other populations.

**Figure 2.**
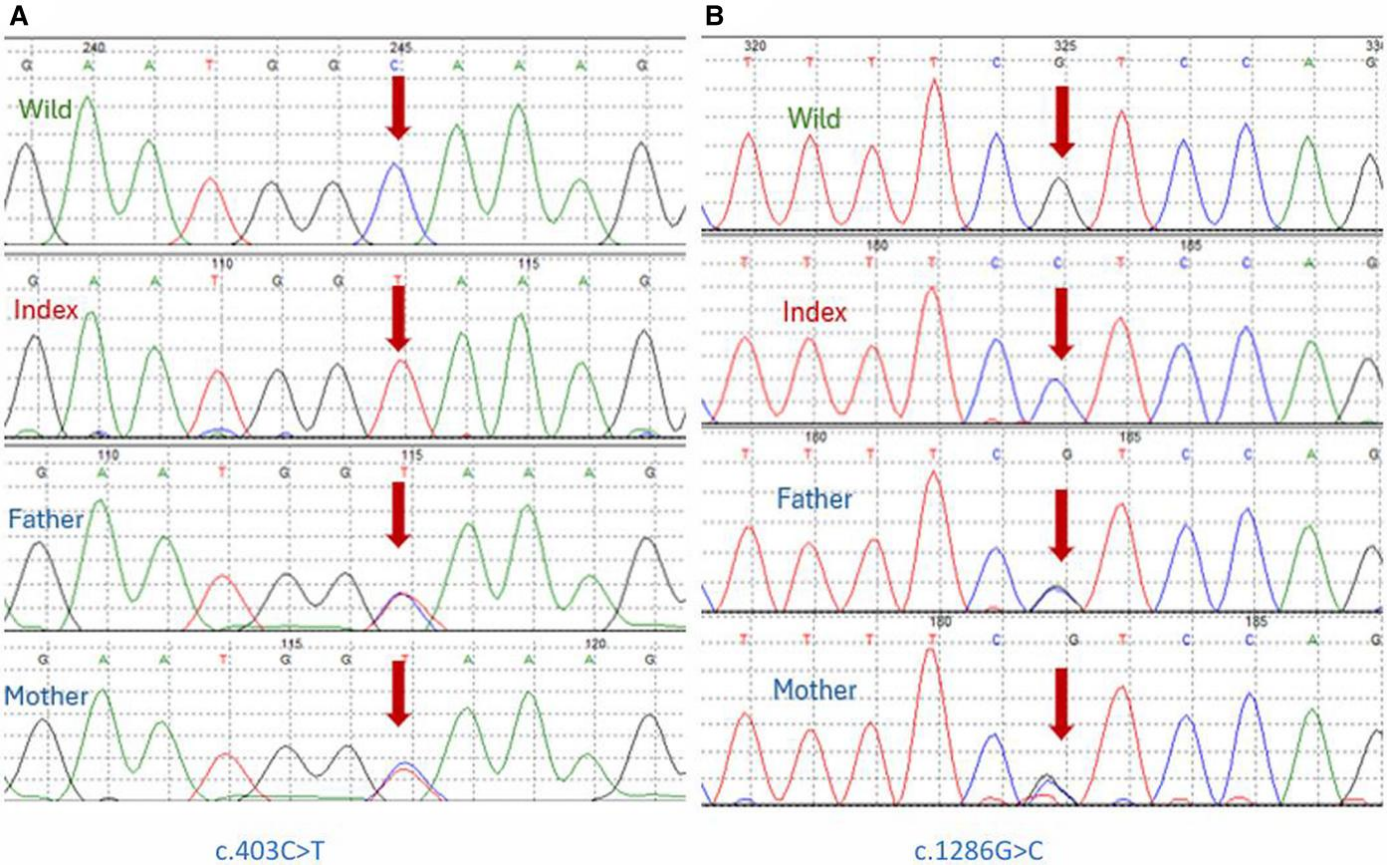
Chromatograms of some *CYP27B1* mutations found in this study showing **A**, the missense mutation c.403C > T, and **B**, the missense mutation c.1286G > C. The upper panel is the wild-type sequence followed by the sequence of the index cases and the sequence of father and mother. Arrows indicate the sites of the mutations.

The mutations found in this cohort are summarized in Table 1. We found 10 different mutations in the 29 families (Fig. 1). These included 5 missense mutations occurring in 32 patients from 20 families, 2 nonsense mutations in 3 patients from 3 families, 2 insertion mutations in 6 patients from 4 families, and 1 splice-site mutation in 4 patients from 2 families (Table 1). Four of these mutations were novel (40%) while 6 were previously described, including 1 that was previously described by our group only (c.305G > A p.(Gly102Glu) and has never been reported from any other population, and 1 (c.1510C > T, p.(Gln504Ter) that was reported from Saudi Arabia as a novel mutation and has never been reported again in the literature (Fig. 1 and Table 1). This increases the rate of novel/previously novel and never been reported again to 6 of 10 mutations (60%). The most common mutation was c.1286G > C, p.(Arg429Pro) occurring in 22 patients from 15 unrelated families (51.7%) (Fig. 2). Six of these families had 2 or 3 members affected, and 9 patients were single patients from different families (Table 1). The insertion/frameshift mutation c1319_1325 dupCCCACCC, p.(Phe443Profs*24) was the second most common, occurring in 3 families followed by the missense and splice-site mutations, c.1192G > A, p. (Gly398Ser) and c.589 + 1G > A, occurring in 2 families each. The other mutations occurred in 1 family per mutation (Table 1).

**Table 1.** *CYP27B1* mutations found in the present study

Nucleotide change (NM_000785.4)	Amino acid change	Novelty	Families, >1 patient affected*^a^*	Families, a single patient affected*^a^*	Total Nos. of families (patients)
c.1286G > C	p.(arg429Pro)	Reported once	2, 2, 2, 2, 3, 2,	1, 1, 1, 1, 1, 1, 1,1, 1	15 (22)
c.1319_1325dupCCCACCC	p.(Phe443Profs*24)	Reported	2, 2	1	3 (5)
c.403C > T	p.(Gln135Ter)	Reported	—	1, 1	2 (2)
c.589 + 1G > A		Reported	2, 2	—	2 (4)
c.305G > A	p. (Gly102Glu)	Reported once from KSA	2	—	1 (2)
c.1510C > T	p.(Gln504Ter)	Reported once from KSA	—	1	1 (1)
c.768dup	p.(Trp257LeufsTer76)	Novel	—	1	1 (1)
c.301G > C	p.(Glu101Gln)	Novel	—	1	1 (1)
c.1192G > A	p.(Gly398Ser)	Novel	2, 4	—	2 (6)
c.616C > T	p.(Arg206Cys)	Novel	_	1	1 (1)
Total			29 patients (13 families)	16 patients (16 families)	29 families (45 patients)

Abbreviation: KSA, Kingdom of Saudi Arabia.

^a^Each number represents a single family with the number of patients affected.

### Novel Mutations in the Present Study

**Figure 3.**
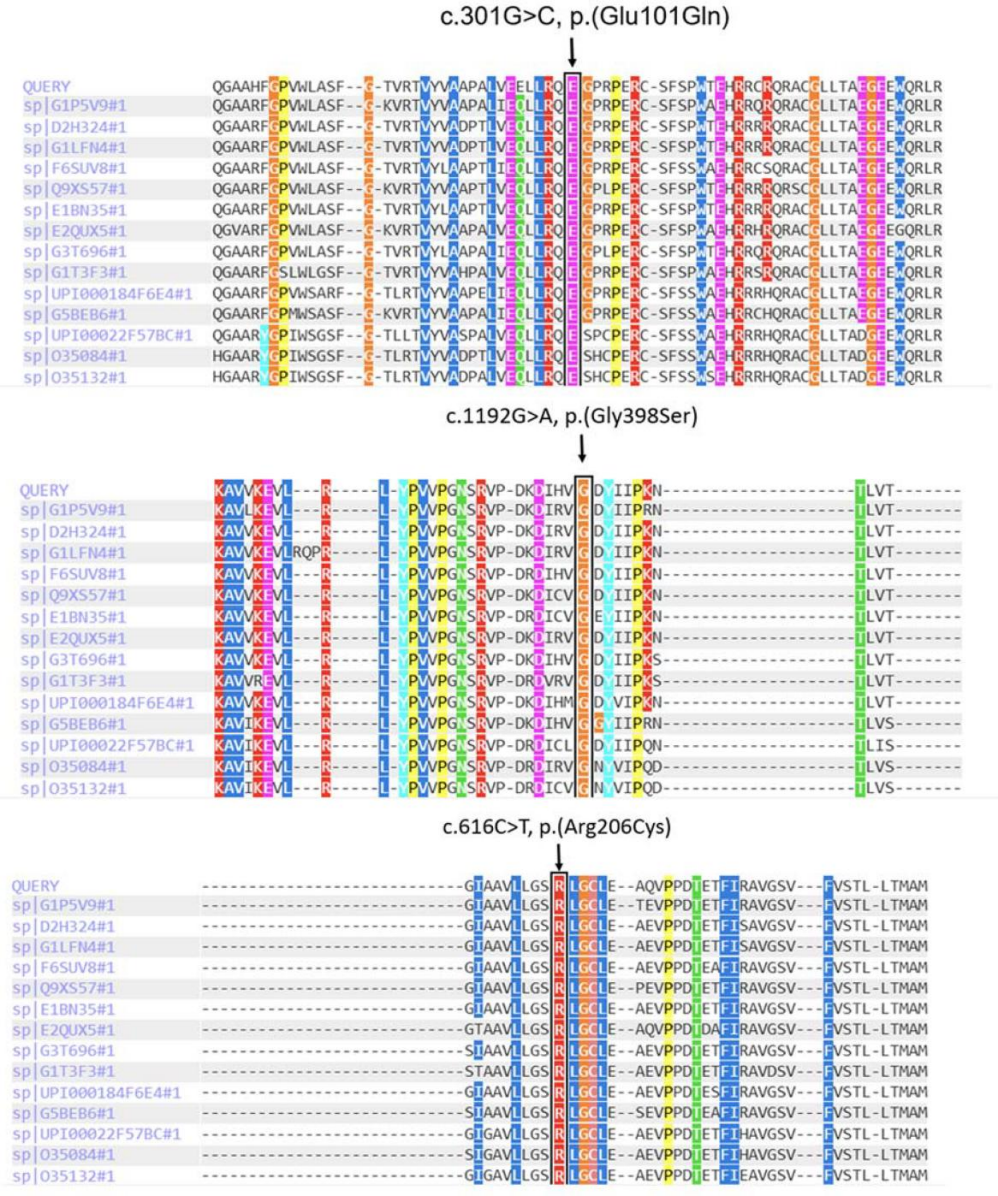
Multiple sequence alignment among different species. The novel variants are highly conserved across different species.

Four variants found in this study are novel (Table 2). In silico analysis of these mutations is shown in Table 2. c.768dup,p.(Trp257Leufs*76) is an insertion mutation that leads to frameshift and truncation of 76 amino acids downstream and is expected to disrupt the protein structure and function. It is characterized as pathogenic by the American College of Medical Genetics and Genomics (ACMG) classification (Table 2). The c.301G > C, p.(Glu101Gln) mutation found in 1 patient in the present study is also a novel one. It is characterized as a pathogenic variant by PolyPhen-2 (probably damaging with a score of 1.0), and as deleterious by MutationTaster and SIFT (Table 2). Similarly, the c.1192G > A (p.Gly398Ser) variant is a novel one found in a family with 2 affected siblings and is characterized as deleterious by MutationTaster and SIFT and as benign by PolyPhen-2 and AlphaMissense. It is not found in common population databases and is characterized as a variant of unknown significance (VUS) by EVE and ACMG classification (Table 2). Finally, the missense variant c.616C > T, p.(Arg206Cys) is a novel mutation found in 1 patient. It is predicted to be damaging by PolyPhen-2, deleterious by SIFT and VUS by AlphaMissense and ACMG classification. All these variants were not found in ExAC, 1000G, or gnomAD and are highly conserved among different species (Fig. 3)

**Table 2.** In silico prediction of 4 novel *CYP27B1* variants found in the present study

Variants	Allele frequency (ExAC, 1000G and GnomAD)	MutationTaster	SIFT	PolyPhen-2	EVE*^a^*	AlphaMissense	ACMG classification
c.768dup, p.(Trp257Leufs*76)	Not found	Deleterious					Pathogenic
c.301G > C, p.(Glu101Gln)	Not found	Deleterious	Deleterious	Probably damaging	VUS	VUS	VUS
c.1192G > A, p.(Gly398Ser)	Not found	Deleterious	Deleterious	Benign	VUS	Benign	VUS
c.616C > T, p.(Arg206Cys)	Not found	Benign	Deleterious	Probably damaging	Pathogenic	VUS	VUS

Abbreviations: ACMG, American College of Medical Genetics and Genomics; EVE^a^, Evolutionary Model of Variant Effect; SIFT, Sorting Intolerant From Tolerant; VUS, variant of unknown significance.

### Previously Reported *CYP27B1* Mutations From Saudi Arabia

To further understand the genotype of *CYP27B1* in Saudi Arabia, we also searched and summarized all cases of 1α-hydroxylase deficiency with *CYP27B1* mutations reported in the literature from Saudi Arabia (Table 3). Only 6 reports (7 patients) were found; 4 of them reported novel *CYP27B1* mutations in single patients (Table 3) that have never been reported again from any other populations (Table 3). Two of these previously reported novel mutations (c.305G > A and c.1510C > T) were also found in patients in this study (Table 1).

**Table 3.** Previously reported *CYP27B* (NM_000785.4) mutations in Saudi patients with 1α-hydroxylase deficiency

Nucleotide change	Amino acid change	No. of patients/families	Novelty when reported	Reported again	Reference
c.305G > A	p.Gly102Glu	6/1	Yes*^a^*	No	Alzahrani et al [19]
c.373G > A	p.Gly125Arg	1/1	Yes	No	Dubaee et al [34]
c.403C > T	p.(Gln135Ter)	1/1	No	Yes, 1 report	Monies, et al [28]
c.398_400dupAAT	p.(Trp134Ter)	1/1	Yes	No	Durmaz et al [22]
c.1319_1325dupCCCACCC	p.(Phe443Profs*24)	1/1	No	Yes, many reports	Durmaz et al [22]
c.1375C > T	p.(Arg459Cys)	1/1	No	Yes, many reports	Al Homyani et al [33]
c.1510C > T	p.(Gln504Ter)	1/1	Yes*^a^*	No	Babiker et al [20]

^a^These mutations were again found in the present study.

## Discussion

In this study, we have analyzed the molecular genetics of 1α-hydroxylase deficiency, the cause of type 1 rickets, in a large cohort from the highly consanguineous population of Saudi Arabia. The gene for this enzyme is located on chromosome 12q14.1 and consists of 9 exons, 509 codons, and 1527 nucleotides [24, 25]. Our analysis revealed a number of mutations of different types distributed throughout the *CYP27B1* gene. Missense mutations were the most common (50%) but there were also nonsense (20%), insertions with frameshift (20%) and splicing-site mutations (10%). The missense c.1286G > C p.(Arg429Pro) mutation was the most common mutation, occurring in 22 patients of 15 unrelated families (∼52%). This suggests that this is a founder mutation in the Saudi population. Although one of the earliest described mutations in *CYP27B1*, this mutation had been described only once in a Black American child by Wang et al in 1998 [10] with no further reports since then. Its functional analysis showed no 1α-hydroxylase activity confirming its pathogenicity. The c.305G > A (p.Gly102Glu) missense mutation was also described and functionally characterized only once by our group in a single family with 6 affected members [19]. In the present study, we found the same mutation in another unrelated family with 2 affected siblings. This also suggests that this mutation predominates in our population.

The c1319_1325 dupCCCACCC (p. Phe443Pro fs*24) mutation was found in 5 patients from 3 families in this study. It has been reported in several studies including a study of ours of 7 Turkish and 2 Saudi families [26]. In that study, this variant was present in 1 Saudi and 5 Turkish families. The current patients who carry this mutation are not related to the previously reported Saudi family.

The nonsense mutation c.403C > T, p.(Gln135Ter) was found in 2 patients from 2 unrelated families. It was previously reported in a Turkish patient [27] and as part of a report on a large scale-sequencing database from Saudi Arabia [28]. We could not confirm or rule out if the current patient is the same or a relative of the previously reported patient, as we did not have access to the data of the previous report. Similarly, the splicing-site mutation (c.589 + 1G > A) was previously reported in at least 4 studies [29-32] and 1 of them was from Saudi Arabia [31]. Again, we could not ascertain whether the patient included in the previous study was the same or a relative of the current ones. In the present study, this mutation was found in 4 patients from 2 unrelated families.

The present study shows 4 of 10 novel mutations (40%). The c.768dup, c.301G > C, p.(Glu101Gln, c.1192G > A, (p.Gly398Ser), and c.616C > T, p.(Arg206Cys) are all novel mutations not found in the major population databases such as ExAC, 1000G, and gnomAD and are predicted by most in silico analysis tools to be deleterious to the protein function (Table 2). In addition to the 4 novel mutations found in the present study, 4 of 7 previously reported mutations from Saudi Arabia were novel and remained novel as no further reports from other populations have published these mutations (Table 3). Two of them were found in new families included in the present studies.

Collectively, the genotype of 1α-hydroxylase deficiency (*CYP27B1*) in Saudi patients is unique with a predominance of c.1286G > C (p.arg429Pro) mutation and a number of novel or rarely reported mutations. Most of the previously reported mutations found in this cohort were also from Saudi patients and never been reported from other ethnic groups, confirming the unique genotype of *CYP27B1* deficiency in our population. We previously reported 6 siblings of a single Saudi family who had a novel *CYP27B1* mutation (c.305G >A) and presented with substantial biochemical abnormalities, short stature, and severe skeletal deformities [19]. This mutation has never been reported again from any other population and is found in 2 siblings of 1 family in the present study. Babiker et al [20] reported a 13-month old Saudi girl with severe manifestations of 1α-hydroxylase deficiency due to a novel *CYP27B1* truncating mutation (p.Gln504Ter). This mutation has also never been reported again and is found in one family of the present cohort. Al Homyani and Alhemaiani [33] reported a 4-year-old Saudi boy with very severe disease and another truncating *CYP27B1* mutation, p.(Arg459Cys). This has not been reported again from any other population. Dubayee et al [34] reported a 17-month-old Saudi girl who had been suffering from motor developmental delay, delayed dentition, and macrocephaly who was found to have 1α-hydroxylase deficiency and a missense *CYP27B1* mutation (p.Gly125Arg) that has also never been reported again. Our study and these previous case reports show a unique genotype of *CYP27B1* in the Saudi population. In total, out of 17 *CYP27B1* mutations reported in this study (10 mutations) and previous reports from Saudi Arabia (7 mutations), 8 (47%) are novel mutations (4 in this study and 4 in previous reports). This is likely due to the high consanguinity and the homogeneous population of Saudi Arabia [13, 14]. In a country-wide study, El-Hazmi et al [13] interviewed more than 3000 families and found the overall rate of consanguinity to be 57.7%, with regional variations ranging from 52.1% to 67.7%. El-Mouzan et al [15] performed a national survey a decade later and reported similar rates, with overall rates of consanguinity of 56% and rate of first-degree cousin marriages of 33.6%. In a more recent survey of 600 Saudi women about their family structure and consanguinity, the prevalence of consanguinity was 52%; the authors concluded that consanguinity rates have not changed over a generation and within-family marriages are still preferred in Saudi society despite improved awareness of the risk of genetic diseases with consanguinity [35]. These high consanguinity rates contribute considerably to the increased risk of genetic diseases as reported in a number of studies from Saudi Arabia [28, 36-39].

Our study is the first to characterize the molecular genetics of a large cohort of patients with *CYP27B1* mutations from the Arab world. It reveals a unique genotype with several novel mutations and a number of previously described mutations. Most of the previously described mutations found in this study were described solely from Saudi Arabia, attesting further to the unique genotype of 1α-hydroxylase deficiency in this country. However, our study also has some limitations, including the inability to study the genotype-phenotype correlation as the patients have a wide age range and the manifestations are likely age related. In addition, data on the patients were collected from different institutions across the country with variable practice and inconsistent approaches to management. Therefore, we elected not to study genotype-phenotype correlations to avoid reaching incorrect conclusions. However, informative data on the clinical, biochemical, and radiological features of each patient are reported in Supplementary Table S1 [23]. Although we ascertained the nonrelatedness of the families by carefully studying the family history, we cannot exclude distant relationships between some families as consanguinity and cross-marriages are common in Saudi Arabia, and the population structure consists mostly of tribal groups [13, 14]. This may have led to some founder effect of some mutations, especially c.1286G > C, p.(Arg429Pro), which was present in almost half of the cohort although it was previously described only once in the literature.

In summary, this study characterizes a unique genotype of 1α-hydroxylase deficiency in the highly consanguineous population of Saudi Arabia, reporting a large number of novel mutations, a possible founder mutation, and additional previously reported mutations, cited only from Saudi Arabia. These findings might be representative of the underlying molecular genetics of 1α-hydroxylase deficiency in the Arab world and add to the repertoire of novel *CYP27B1* mutations.

## Data Availability

Original data generated and analyzed during this study are included in this published article or in the data repositories listed in “References.”

## Abbreviations

ACMG: American College of Medical Genetics and Genomics
VUS: variant of unknown significance
